# From microbes to mind: germ-free models in neuropsychiatric research

**DOI:** 10.1128/mbio.02075-24

**Published:** 2024-08-29

**Authors:** Susana Delgado-Ocaña, Santiago Cuesta

**Affiliations:** 1 Department of Cell Biology and Neuroscience, Rutgers the State University of New Jersey, Piscataway, New Jersey, USA; The Ohio State University, Columbus, Ohio, USA

**Keywords:** germ-free, gut microbiome, gut–brain axis, neuropsychioatric disorders

## Abstract

The gut–microbiota–brain axis refers to the bidirectional communication system between the gut, its microbial community, and the brain. This interaction involves a complex interplay of neural pathways, chemical transmitters, and immunological mechanisms. Germ-free animal models have been extensively employed to investigate gut–microbiota–brain interactions, significantly contributing to our current understanding of the role of intestinal microbes in brain function. However, despite the many benefits, this absence of microbiota is not futile. Germ-free animals present physiological and neurodevelopmental alterations that can persist even after reconstitution with normal microbiota. Therefore, the main goal of this minireview is to discuss how some of the inherent limitations of this model can interfere with the conclusion obtained when using these animals to study the complex nature of neuropsychiatric disorders. Furthermore, we examine the inclusion and use of antibiotic-based treatments as an alternative in the research of gut–brain interactions.

## INTRODUCTION

Growing evidence supports the importance of the intestinal microbiota for the development and function of the central nervous system. This gut–microbiota–brain axis represents a bidirectional communication system between the gut, its bacterial community, and the brain and involves a complex interplay of neural pathways, chemical transmitters, and immunological mechanisms (1–3). Understanding the intricacies of this axis is crucial for revealing the impact of gut microbiota on brain function and behavior.

Much of our current knowledge regarding the role of intestinal microbiota in brain development, neurochemistry, and neurological functions has been possible, thanks to the development and use of germ-free animals, which provide a powerful approach to investigating microbiota–host interactions. However, despite the many benefits of these germ-free animals, some limitations should be considered when interpreting the results collected using this model, even more so when investigating the microbiota–gut–brain axis in the context of neuropsychiatric disorders. The environment has a critical impact on brain development, and because of its condition, germ-free animals do not completely replicate the complex interactions between the microbial communities and the host found during development. This absence of microbiota is not futile and indeed strongly affects neurodevelopment, from changes in the maturation of neural circuits to neurotransmitter levels and behaviors (4, 5). This intrinsic limitation of the germ-free animals can sometimes interfere with the interpretation and conclusions made from the data collected during their use in experimental setups.

To avoid some of these complexities, broad-spectrum antibiotics have been used as an alternative to deplete the host microbiota. In contrast to germ-free, antibiotics can diminish bacterial populations at different time points during life, avoiding impairments in development and early immune maturation observed in germ-free animals. Diverse antibiotic regimens are frequently employed, varying in composition, dosage, treatment duration, and administration method. This treatment flexibility enables for a temporally restricted reduction or decrease in most microbiota members (6, 7). However, antibiotic treatment also poses several limitations including off-target effects and the potential of developing antibiotic resistance, which need to be considered in the experimental design when using this experimental strategy.

In this minireview, we will go over some of the main changes that have been observed in the physiology of germ-free animals and that have helped reveal and understand the many roles of the gut microbiome in host physiology and homeostasis and more specifically in brain development and function. We will also cover some of the limitations that can arise from the use of this model as a strategy to understand the biological bases underlying different psychiatric conditions. Finally, we will discuss the inclusion and use of antibiotic-based treatments as an alternative in the research of gut–brain interactions.

## THE GUT MICROBIOME

The gut microbiome is a complex ecosystem of microorganisms, including archaea, viruses, fungi, and bacteria, which reside in the gastrointestinal tract of humans and other animals (8). Bacteria are the most prominent population, with over 95% located in the large intestine (9). The adult human body contains trillions of bacteria cells and about 3.3 million distinct bacterial genes (9), roughly 100 times more genes than those in the human host (10, 11). The gut microbiome plays a crucial role in host health, significantly affecting digestion, metabolism, immune function, and neurological processes (12).

The origins of the gut microbiota can be traced back to birth, typically derived from the mother, with the initial colonization taking place during delivery and continuing throughout early infancy (13). This seeding process largely depends on several factors, including the birth delivery mode, diet, antibiotic exposure, habits, and even the individual’s genetics (14–17). This early exposure to microbes leads to diverse microbial ecosystems in the skin, mouth, vagina, and lungs, but the majority of these bacteria reside in the lower gastrointestinal tract (18).

After its establishment during early adulthood, each person will harbor a unique and individualized microbiome composition (19, 20) that will include Firmicutes and Bacteroidetes as the most abundant bacteria phyla and Actinobacteria and Proteobacteria as lower-proportion constituents (19, 21). While stable, this adult microbiome is not static and similar to its establishment can be altered by many environmental factors and experiences, returning to a similar state of equilibrium after cessation of these external insults (22). However, these transient variations can modify normal gut physiology and metabolite production, leading to changes in homeostasis, metabolisms, and gene expression, which can result in long-lasting effects on the host (19, 23). In fact, alterations in the quantity and composition of the microbiome are associated with disorders of the immune, endocrine, and nervous systems (24).

## DEFINITION OF GERM-FREE AND CURRENT METHODS OF GENERATION

A germ-free animal refers to an animal that is completely devoid of microorganisms, including bacteria, viruses, fungi, and parasites, both internally and externally (25). This germ-free state is typically achieved through stringent aseptic techniques and regular monitoring and testing to ensure the absence of any detectable microbial contaminants (6, 25). The origin of the “germ-free” concept can be attributed to Louis Pasteur in 1885, who hypothesized that bacteria were vital for animal survival because of the co-evolution between microbes and their hosts (26, 27). In 1897, the first germ-free guinea pigs were generated (28), but it was not until the 1950 s that several authors were able to simultaneously generate successive germ-free rodents (29–32).

Currently, the methods for generating germ-free rodents include performing aseptic Cesarean sections on pregnant animals, ensuring no contact between the neonates and their mother, and immediately transferring the neonates to sterile isolators where they will be maintained in a controlled environment with sterilized food, water, and bedding (33). Strict protocols are implemented to prevent microbial exposure, including equipment sterilization and aseptic animal care practices. Continuous monitoring ensures the absence of contamination through regular microbial screenings and body site sampling (34). Subsequent germ-free animal generations can then be bred in an isolator. Alternatively, these animals can be produced by embryonic transfer at the two-cell stage into a germ-free host mother (35).

## CONSEQUENCES OF GROWING UP GERM-FREE

**Fig 1:**
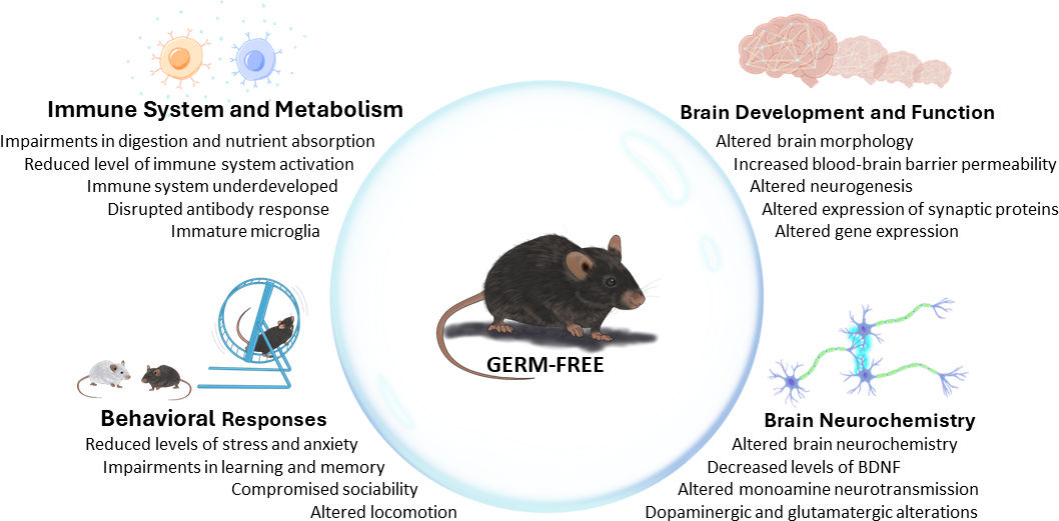
Microbiota is essential for host homeostasis and physiology. Germ-free mice have impairments in the immune system and metabolism and alterations in brain function, neurochemical signals, and behavior.

Studies on germ-free animals have shown that while animals can survive lacking a microbiota, its absence is associated with abnormal development, physiology, and immunology affections. The microbiota is essential for maintaining normal host homeostasis, and growing up germ-free leads to significant changes in the host’s body weight (5, 36), intestinal function (5), immune system responses (37, 38), life span (39–41), hormone signaling, metabolism, and brain function (33) (Fig. 1).

### Immune system

The immune system is constantly developing from conception onward, and the gut microbiota has been shown to play a crucial role in this process (42, 43). Germ-free animals exhibit an underdeveloped immune system and reduced levels of immune system activation compared to conventionally raised animals (8, 44). The absence of microorganisms results in a lack of immune challenges, such as pathogens or commensal bacteria, that normally stimulate the immune system (8). As a result, germ-free mice have an overall reduction in the numbers and an altered proportion of immune cells (37, 45) and present a diminished presence of macrophages, mast cells, and mucosal-associated invariant T cells in the intestine (45–50). Additionally, innate immune cells within the gut display an immature phenotype, wherein macrophages lack the hyporesponsiveness to lipopolysaccharide (LPS) needed for commensal microbiota tolerance (45, 51). Furthermore, innate lymphoid cell type 3 (ILC3) exhibits an inability to produce the required levels of interleukin-22 (IL-22) essential for host defense, even upon stimulation (52, 53). Similarly, after splenocyte stimulation with lipopolysaccharide, the production of the proinflammatory cytokine tumor necrosis factor α (TNFα) is blunted in germ-free mice (54). Due to these changes, the ability of germ-free animals to manage microbial colonization and their response to infection and tissue damage is compromised, as indicated by the decreased expression of antimicrobial peptides at barrier sites (52, 55, 56).

Antibody-mediated responses are also disrupted in germ-free mice, with fewer IgA-producing plasma cells populating the gut-associated lymphoid tissues (GALT), lamina propria, lung, and bone marrow, and reduced circulating levels of all antibody classes, except for IgE, which is pathologically elevated (45, 57, 58). Furthermore, in comparison to conventionally housed animals, germ-free animals have reduced expression of activation markers on intestinal macrophages, decreased major histocompatibility complex class II (MHCII) on epithelial cells, and lower nitric oxide and histamine levels in the small intestine (37, 59–63). At the level of the brain, the immune system is also affected in these mice. When compared with conventional animals, germ-free mice have increased numbers of immature microglia in several brain regions and decreased microglial responsiveness, revealed by alterations in cell morphology and transcriptional markers of maturity in microglia cells. In response to a bacterial or viral challenge, these microglia have reduced proinflammatory gene expression and do not show the typical activated morphology (64, 65). Altogether, these alterations result in a compromised immune response and increased susceptibility to infections.

### Organ development and metabolism

The gut microbiome also impacts the development of diverse organs: germ-free animals present a smaller and less developed thymus than conventionally raised animals (38, 66), and the maturation signals for GALTs are absent, leading to smaller Peyer’s patches and mesenteric lymph nodes showing reduced size (67–69).

Germ-free mice also have an altered gut morphology, showing a reduction in total intestinal mass, enlarged cecum, thinner villi, and mucus layers; reduced epithelial cell renewal; and slower intestinal motility (45, 70–75). These animals present impairments in digestion associated with altered gastrointestinal enzyme content and impaired nutrient absorption (34, 70, 76, 77). As a consequence of these changes, germ-free mice are thinner and have less body fat than normal mice, require a higher caloric intake to maintain the same body weight than conventional animals, and, since they are prone to vitamin deficiencies, they need dietary supplementation with vitamins K and B (4, 36, 70, 78, 79). Growing up in the absence of microbes has negative effects on the production of gut metabolites as short-chain fatty acids (SCFAs), which are normally generated through the fermentation of dietary fiber by bacterial communities in the gut and play a crucial role in supporting host energy metabolism (5, 80). These altered gut microbiota-derived metabolites can affect neurotransmitter production, neuroinflammation, and blood–brain barrier integrity and have been linked to neurodegenerative, neuroinflammatory, and neuropsychiatric disorders such as Alzheimer’s disease (AD), Parkinson’s disease (PD), and autism spectrum disorders (ASD) (65, 81).

### Brain development and neuronal function

The gut microbiome plays an essential role in various processes of brain development and function such as neurogenesis, myelination, microglial maturation, development and maintenance of blood–brain barrier integrity, and in the hypothalamic–pituitary–adrenal axis maintenance (82).

Many differences in brain morphology have been reported in germ-free animals. Both the amygdalar and the hippocampal volume of the cornu ammonis region 2 and 3 (CA2/3) are increased in these mice compared to specific pathogen-free (SPF) mice (83). However, the germ-free status has different consequences in dendritic morphology in the basolateral amygdala and hippocampus. In the basolateral amygdala, germ-free animals show dendritic hypertrophy and increased spine density. In contrast, in the hippocampus, the absence of a gut microbiota induced dendritic atrophy in both pyramidal neurons and dentate granule cells and a loss of stubby and mushroom spines on hippocampal pyramidal neurons, indicating that the microbiota plays a crucial role in maintaining the typical structure and detailed organization of the amygdala and hippocampus (83). Germ-free mice also show lower levels of myelin-related proteins and less mature oligodendrocytes compared with SPF mice (84).

The blood–brain barrier is a highly selective and protective barrier that separates the blood circulation from the brain’s central nervous system and regulates the passage of substances between the bloodstream and the brain tissue (85). This barrier is more permeable in germ-free mice and presents reduced expression of the tight junction proteins occludin and claudin-5, which are crucial for the regulation of barrier function (86). This augmented brain permeability would lead to an increase in the diffusion of molecules into the growing brain and could be one of the mechanisms behind the alterations observed in neural connectivity, physiology, and behavior in these mice (86).

The gut microbiota is also essential for the establishment of brain circuitries and synapses. In the striatum, expression of the synaptic proteins synaptophysin and postsynaptic density protein-95 is elevated in germ-free mice (87). This modulation by the gut microbiota has been proposed to lead to long-term modulation of synaptic transmission, affecting motor control and behavior in adult life (87). Additionally, germ-free mice show disruptions in the development and maturation of the dentate gyrus functional network and substantial alterations in functional connectivity and neurogenesis (88). Scott and colleagues (88) also found that the age-related decline of neurogenesis is disrupted in germ-free mice. Notably, this effect varies between sexes; while males show diminished neurogenesis at 4 weeks, females exhibit heightened neurogenesis at 8 weeks (88).

The complete lack of a microbiome in germ-free mice also significantly impairs gene expression in the hippocampus (89), the prefrontal cortex (90, 91), the nucleus accumbens (92), and the amygdala (93, 94). Germ-free mice show a marked dysregulation in the overall transcriptome and synaptic-related gene dysregulation in pathways involved in cellular metabolic processes (92, 94, 95). Furthermore, the absence of gut microbiota also influences the expression of microRNAs involved in metabolic processes and cellular pathways, including axon guidance in the hippocampus (96). In this same region, significant changes in the expression pattern of long noncoding RNAs associated with the regulation of pathways involved in cardiac hypertrophy and signaling pathways involving nuclear factors of activated T cells, gonadotropin-releasing hormone, calcium, and cAMP-response element-binding protein have been reported (89).

### Behavior

In line with the evidence that the absence of a normal gut microbiota can influence brain development, different behavioral alterations have also been reported in these animal models.

#### Stress and anxiety

Studies conducted on germ-free animals have demonstrated that microbiota influences stress reactivity and anxiety-like behavior and regulates the set point for hypothalamic–pituitary–adrenal axis activity (2, 97, 98). In this regard, several investigations have shown that the absence of microbiomes leads to reduced levels of anxiety-like behavior compared to SPF mice (54, 87, 99, 100). This altered behavior is accompanied by changes in the mRNA expression of genes implicated in anxiety and stress reactivity, including decreased N-methyl-D-aspartate receptor subunit NR2B mRNA expression in the central amygdala and decreased serotonin receptor 1A (5HT1A) expression in the dentate granule layer of the hippocampus (99). However, some studies have found different results (101–103), proposing that while the absence of a microbiome would impact this behavioral response, other factors such as age, strain, sex, and behavioral task will also influence the response. Similarly, locomotion is altered in germ-free mice, and this change would be sex-dependent, with males displaying increased locomotor activity and rearing (87, 104) and females not showing any alteration in these behaviors (99).

#### Learning and memory

Gut microbes seem to modify the ability to form memory. Studies have revealed significant alterations in learning and memory processes in germ-free mice, suggesting an important role of the gut microbiota in cognitive functions (105). Gareau and colleagues (101) revealed that germ-free mice exhibited a decrease in both non-spatial and working memory when exposed to either a novel object or a T-maze (101). A reduction in c-Fos positive cells in the hippocampus was observed in these germ-free mice when compared to the control group (101), which could be mediating these deficiencies. Similar findings were observed by Lu and colleagues (106) who described that germ-free mice exhibit deficits in spatial learning and memory tasks, such as the Morris water maze, which is accompanied by morphological changes in the brain, such as a decrease in striatum volume (106). Altogether, these data support an impaired and compromised ability to form and consolidate spatial memories in germ-free mice.

#### Sociability

Social behavior is also affected in germ-free mice. Although the results differ, most studies indicate that germ-free mice exhibit impaired social activity (106–108). Desbonet and colleagues (107) showed that germ-free animals exhibit disruptions in both social preference (germ-free mice spend more time exploring an empty chamber than a chamber containing a mouse) and social recognition (germ-free mice fail in identifying a novel over a familiar mouse) (107). Similarly, Lu and colleagues (106) showed that compared with SPF, germ-free mice have disrupted social cognition, spending significantly less time with a novel mouse than a familiar mouse, but in the social preference test, no significant differences between these groups were observed (106). In contrast, Arentsen and colleagues (100) found that germ-free mice display increased social preference compared to conventionally raised mice (100). These apparent discrepancies between the studies would be due to differences in experimental design since different mice strains and ages were used (100).

### Brain neurochemistry

Although the molecular mechanisms underlying how the microbiota modifies host behavior are still being established, evidence suggests the microbiota alters the neurophysiology of brain areas considered key nodes for social and anxiety behavior networks, including the prefrontal cortex, amygdala, and hypothalamus (105). Brain-derived neurotrophic factor (BDNF) is a protein that plays a critical role in the development, function, and plasticity of the nervous system (109). In germ-free mice, BDNF expression is reduced in the cortex and the amygdala compared to controls (87, 100). Similarly, in the hippocampus, most of the studies demonstrated that germ-free mice exhibit a decrease in the expression of this neurotrophic factor (87, 98, 101). As an exception, an increase in BDNF expression in the dentate gyrus of the hippocampus of germ-free female Swiss Webster mice has been reported (99).

Monoamine neurotransmission is also affected in germ-free animals. Heijtz and colleagues (87) showed that these animals present an increase in the turnover rate of noradrenaline (NA), dopamine (DA), and serotonin (5-HT) in the limbic system, a change that could potentially mediate the differences in anxiety-like behaviors observed in these animals (87). Dopaminergic and glutamatergic synapsis pathways are also affected in germ-free mice, with increases in the dopamine receptor subunit Drd1a in the hippocampus (87) and changes in the expression of the Nr1 and Nr2 glutamate receptor subunits in the cortex, hippocampus, and central nucleus of the amygdala (98, 99). Disruptions of neuropeptides implicated in social behaviors have also been observed in these animals (100, 107, 110).

While more research is needed to describe the molecular mechanisms by which the gut microbiome modulates brain neurochemistry, a possible link between gene expression and metabolomic profiles has been proposed (90). This is mostly based on the observation that cFOS mRNA expression is significantly correlated with several key brain metabolites that are significantly altered in the germ-free phenotype, including glutamate and glutamine (90).

## GERM-FREE MODELS AND NEUROPSYCHIATRIC RESEARCH

The use of germ-free models has allowed to establish the causality between specific bacteria and observed phenotypes in different pathological setups. Through the introduction of defined bacterial strains*—*gnotobiosis— or complex native microbiotas using fecal microbiota transplantations (FMT) into germ-free animals, it has been possible to systematically investigate the direct impact of individual microbes on host phenotypes, enabling a more precise understanding of the causal relationship between specific bacteria and observed biological outcomes in many different contexts, including the microbiota–gut–brain axis (111, 112). Germ-free models have also been essential for understanding bacteria–metabolite–brain interactions, offering unique insights into how microbial metabolites influence nervous system development and function (90, 113–116). However, it is worth noting that consistent evidence has demonstrated that growing up without a microbiota leads to changes in the host immune system and metabolism and, particularly, in brain development, neural function, behavior, and neurochemistry. Therefore, it is crucial to consider and be mindful of the limitations of this model when using it in the study of brain disorders.

Neuropsychiatric conditions encompass a group of multifactorial medical conditions that result from intricate interactions between many factors, including the individual neurobiology, biological sex, and genetic profile, as well as many other environmental and socioemotional conditions (117). These disorders are often associated with disruptions in cognition, emotion, behavior, perception, and personality changes (118) and involve complex neurochemical imbalances, alterations in neural circuitry, and dysregulation of neurotransmitter systems (119). The vast majority of animal models used in preclinical research are based on the measurement of behavioral parameters such as learning, memory, locomotor activity, and social behavior, as well as the assessment of biochemical parameters, such as the levels of specific neurotransmitters like the serotonergic and dopaminergic systems, and factors like BDNF (120).

**Fig 2:**
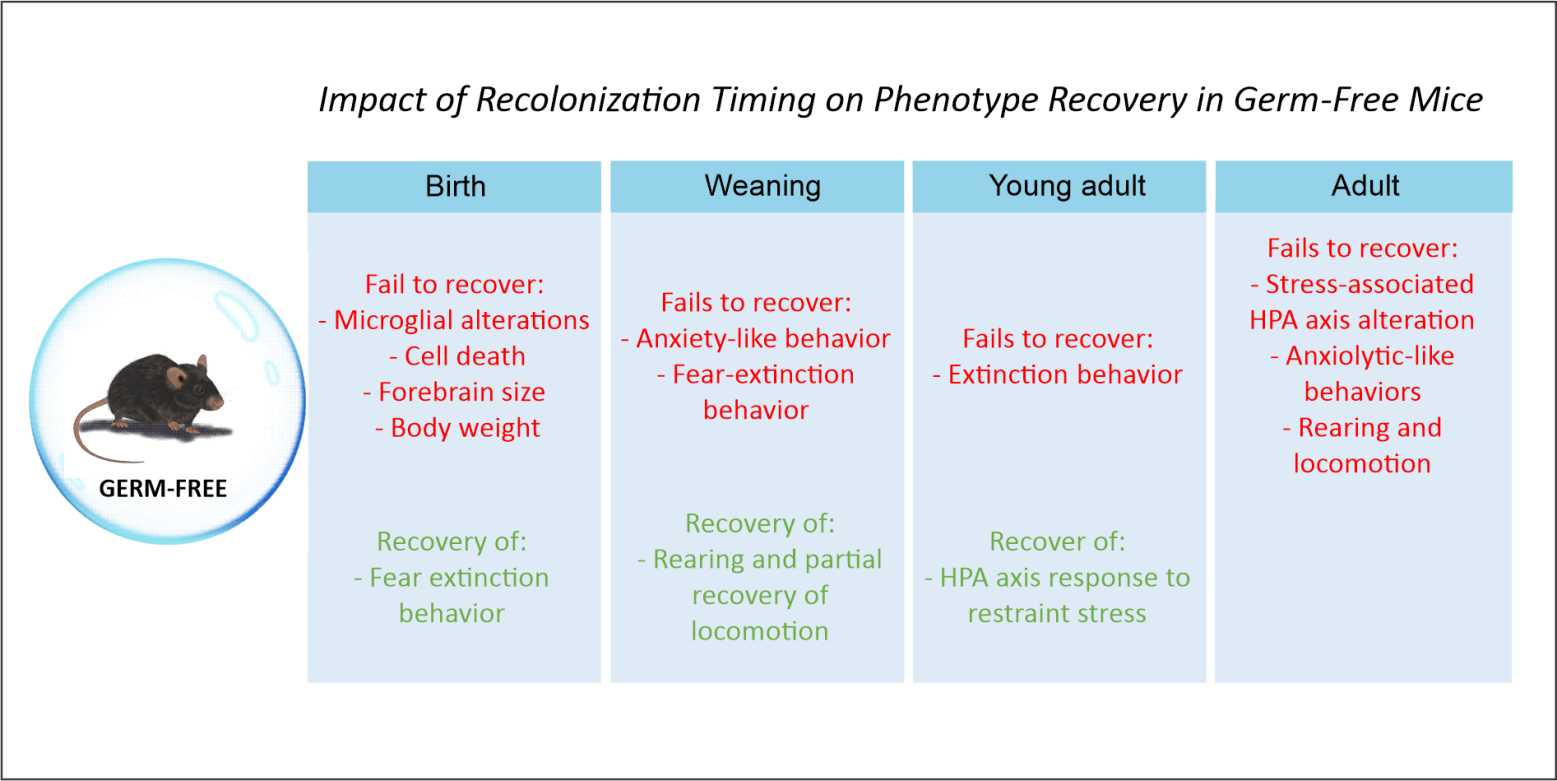
Impact of recolonization timing on phenotype recovery in germ-free mice. Reintroduction of the gut microbiota during the postnatal period or during adulthood results in a limited recovery of functional and behavioral alterations.

Growing under germ-free conditions involves significant changes in many different systems and impacts the animal’s behavior and physiology (Fig. 1). The inherent limitations of this model require extreme caution when interpreting experimental results as they may be influenced by the bias imposed by the germ-free condition. In this regard, it is important to note that the reintroduction of microbiota during the postnatal period or during adulthood has resulted in a limited recovery of functional and behavioral alterations (87, 95, 121, 122), indicating a level of irreversibility of certain consequences related to early-life microbial perturbations (Fig. 2). Recently, Castillo-Ruiz and colleagues (121) have shown that the alterations observed in the brain development of newborn mice gestated and born germ-free persists during the first postnatal week, despite microbiota reconstitution at birth (121). Newborn mice gestated and born germ-free have increased microglial labeling and alterations in developmental neuronal cell death in the hippocampus and hypothalamus, as well as greater forebrain volume compared to conventionally raised mice (121, 123). Interestingly, even after full microbiota reconstitution, the germ-free brain phenotype persisted during at least the first 7 days after birth, suggesting that an altered microbial environment during gestation influences and programs neonatal brain development (121). Similarly, Chu and colleagues (95) showed that germ-free animals present altered fear extinction responses and that only recolonization with a complete microbiota from healthy control mice immediately after birth, but not during weaning ages or adulthood, was able to reverse this impairment (95). These data support the idea that microbiota-derived signals influence processes such as extinction learning and learning-related plasticity since early developmental periods and that these alterations cannot be reversed by reconstituting the animals in adulthood (95).

In the same way, anxiety-like behavior and HPA axis activity also remain affected in previously germ-free mice after postnatal colonization (87, 98, 122). Sudo and colleagues (98) found that reconstituting germ-free mice with SPF microbiota at 6 weeks reversed the HPA axis response to stress, but not at 14 weeks (98). Similarly, reconstitution of germ-free mice early in life only reversed anxiety-like behavior and locomotion partially (87). Furthermore, Neufeld and colleagues (2011) showed that adult reconstitution with normal microbiota does not reverse the anxiolytic phenotype observed in germ-free mice (99, 122). Altogether, this evidence shows that gut–brain interactions are important to the neurodevelopment of stress systems and that a critical window may exist after which reconstitution with a normal microbiota does not normalize the behavioral phenotype (122).

Finally, a question that remains unexplored is whether the rearing conditions of germ-free animals have an effect on brain maturation and adult behaviors. While it is largely known that the environment has a strong impact on brain development and behavioral responses (124–126), to the best of our knowledge, there is little evidence regarding whether the controlled environments that were germ-free are raised, exposed to particular stimuli like noise and handling, and influence the normal development of this animals. It is not completely clear how these differences affect the ability of germ-free animals to adapt to “normal” environments post-exposure to microorganisms and other environmental stimuli and raises some questions about the direct generalizability of findings from germ-free studies to real-life contexts and underscores the need for research assessing how animals’ prior history, including rearing conditions, influences their response to subsequent environmental challenges.

## ANTIBIOTIC TREATMENTS AS AN EMERGING METHOD IN GUT-BRAIN INTERACTIONS RESEARCH

An alternative approach that has emerged to mitigate certain complexities associated with the germ-free system involves the utilization of antibiotic treatment to manipulate the gut microbiome. Administering broad-spectrum antibiotics is a frequently employed method to diminish the gut microbiota in mice that can be applied across various mouse genotypes (6). Unlike the germ-free environment, where lifelong sterility is upheld, antibiotics can reduce bacterial populations in mice that were naturally colonized from birth, avoiding some of the developmental complications inherent in being gestated and born without germens. Conversely to germ-free animals, antibiotic treatment in adult mice enables the exploration of the role of certain bacteria in maintaining cell functionality and signaling pathways post-development (6, 127).

The use of antibiotics has been extensively employed to study the gut–microbiota–brain axis, with various treatments and regimens documented (6, 128, 129). The two most commonly used methods for administering antibiotics in mice are oral gavage or via dilution in drinking water (6, 129). These regimens have enabled to selectively reduce the total microbiota with only 4 days of treatment (6, 129, 130). The use of a combination of ampicillin, neomycin, metronidazole, and vancomycin via oral gavage results in the suppression of bacteria growth in fecal pellets in aerobic and anerobic conditions (131). Similarly, a combination of ampicillin, vancomycin, ciprofloxacin HCl, imipenem, and metronidazole administered *ad libitum* in the drinking water for 6–8 weeks results in the suppression of bacterial growth when monitored by turbidity assessment in ileal contents and bacteriologic analysis of colonic feces of mice after antibiotic treatment (130, 132). Antibiotics can also be used to selectively target different microbial groups. For instance, metronidazole and clindamycin can be used to reduce anerobes, vancomycin is effective against Gram-positive bacteria, and polymyxin B targets Gram-negative bacteria (6, 133, 134) (a more extensive review of broad-spectrum antibiotic treatment regimens for modifying gut bacteria can be found in reference 6). However, while antibiotics can significantly reduce microbial populations, they cannot completely remove gut bacteria as in germ-free animals (129).

Despite their advantages, antibiotic treatments also come with certain limitations and considerations. Similar to what is observed in germ-free mice, antibiotic treatment also has a significant impact on host physiology and function and can potentially produce off-target drug effects (6, 135, 136). Prolonged antibiotic treatments can lead to the development of antibiotic resistance and have been associated with shifts in cell populations, signaling pathways, and brain physiological, behavioral, and cognitive function impairments (135, 137, 138). Interestingly, the changes in the host genetics, physiology, and behavior observed in antibiotic-treated animals do not recapitulate the exact phenotype that is observed in germ-free animals, with studies showing different profiles and responses to treatments between both models (92, 110, 139), suggesting important baseline differences between these two models. Despite these limitations and the lack of standardization of the protocols and type of antibiotics used during these experiments, this strategy is an alternative that can help reduce some of the limitations associated with germ-free mice when studying the neurobiology associated with different psychiatric conditions.

## CONCLUDING REMARKS

As the field continues to grow, it becomes clearer that the interactions between the gut microbiome and the central nervous system are highly complex, as the microbiota plays a crucial role in regulating many factors that have strong implications in brain pathologies. In this context, the use of germ-free mice as a model for the study of neuropsychiatric conditions mandates an additional level of attention when planning the appropriate experimental design, where it is crucial to consider what aspects of the disease are to be evaluated and how the inherent alterations of the germ-free system, even after microbiota reconstitution, could affect the outcomes obtained. On the other hand, antibiotic use offers an alternative approach to manipulating the gut microbiota post-birth and development. However, although not in the same manner, extended antibiotic treatments yield physiological and cognitive deficits similar to those observed in germ-free animals. Hence, the use, evaluation, and standardization of shorter treatment protocols become essential, reducing alterations in both animal physiology and behavior, particularly crucial when investigating disorders of the nervous system, such as neuropsychiatric conditions.

The gut microbiome is consistently showing up as a crucial factor for host physiology and brain function, and there is no doubt that stronger tools will be developed. For the time-being, there is no “one model that fits all,” and the use of a comprehensive multidisciplinary approach that controls for systemic factors (animal sex, strain, and age) and includes carefully selected antibiotic approaches or germ-free animals seems to be the more suitable strategy for investigating these complex brain conditions.
